# Fabrication of Three-Dimensional Composite Scaffold for Simultaneous Alveolar Bone Regeneration in Dental Implant Installation

**DOI:** 10.3390/ijms21051863

**Published:** 2020-03-09

**Authors:** Hun-Jin Jeong, So-Jung Gwak, Kyoung Duck Seo, SaYa Lee, Jeong-Ho Yun, Young-Sam Cho, Seung-Jae Lee

**Affiliations:** 1 Department of Mechanical Engineering, Wonkwang University, Iksan 54538, Korea; hunjinjeong312@gmail.com (H.-J.J.); kdseo85@wku.ac.kr (K.D.S.); 2 Department of Chemical Engineering, Wonkwang University, Iksan 54538, Korea; plus38317@wku.ac.kr; 3 Department of Periodontology, College of Dentistry and Institute of Oral Bioscience, Jeonbuk National University, Jeonju 54896, Korea; hotpeppers0414@gmail.com (S.L.); grayheron@hanmail.net (J.-H.Y.); 4 Department of Mechanical and Design Engineering, Wonkwang University, Iksan 54538, Korea

**Keywords:** alveolar bone regeneration, hybrid scaffold, 3D printing, bone graft particle, additive manufacturing

## Abstract

Dental implant surgeries involve the insertion of implant fixtures into alveolar bones to replace missing teeth. When the availability of alveolar bone at the surgical site is insufficient, bone graft particles are filled in the insertion site for successful bone reconstruction. Bone graft particles induce bone regeneration over several months at the insertion site. Subsequently, implant fixtures can be inserted at the recipient site. Thus, conventional dental implant surgery is performed in several steps, which in turn increases the treatment period and cost involved. Therefore, to reduce surgical time and minimize treatment costs, a novel hybrid scaffold filled with bone graft particles that could be combined with implant fixtures is proposed. This scaffold is composed of a three-dimensionally (3D) printed polycaprolactone (PCL) frame and osteoconductive ceramic materials such as hydroxyapatite (HA) and β-tricalcium phosphate (β-TCP). Herein, we analyzed the porosity, internal microstructure, and hydrophilicity of the hybrid scaffold. Additionally, Saos-2 cells were used to assess cell viability and proliferation. Two types of control scaffolds were used (a 3D printed PCL frame and a hybrid scaffold without HA/β-TCP particles) for comparison, and the fabricated hybrid scaffold was verified to retain osteoconductive ceramic particles without losses. Moreover, the fabricated hybrid scaffold had high porosity and excellent microstructural interconnectivity. The in vitro Saos-2 cell experiments revealed superior cell proliferation and alkaline phosphatase assay results for the hybrid scaffold than the control scaffold. Hence, the proposed hybrid scaffold is a promising candidate for minimizing cost and duration of dental implant surgery.

## 1. Introduction

**Figure 1:**
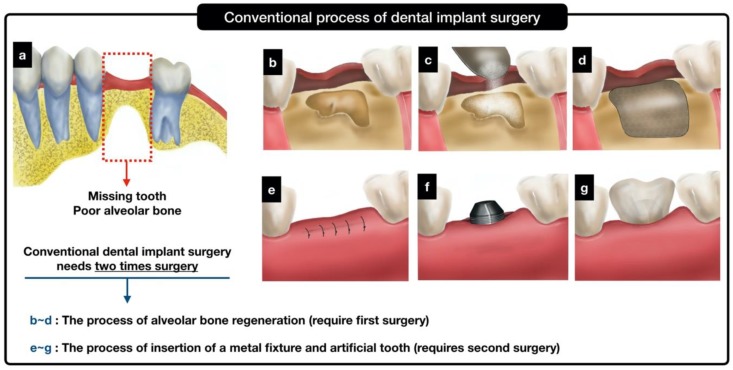
Schematics of a conventional dental implant process and a conceptual process using the proposed hybrid scaffold.

Natural bone graft materials (e.g., autografts, allografts, and xenografts) have been widely used to recover the volumes and shapes of defective bones in edentulous patients with severe alveolar bone defects via regeneration of bones. The gingiva near the missing teeth are first cut to expose the alveolar bone defects, and the bone graft is filled in the defect area. To prevent the loss of bone graft particles, the membrane is covered over the graft, and the flap is sutured. After 3–6 months, the grafted site is reopened, and the implant fixture is installed in the reconstructed alveolar bone. The implant fixture is gradually integrated with the alveolar bone after several months, as confirmed by X-ray examination. Following this, an artificial tooth is inserted at the exposed screw part of the implant fixture, as shown in Figure 1a–g. This complicated treatment usually requires several surgical operations and may cause problems such as patient discomfort, increased treatment time/cost, and increased probability of developing complications [1,2,3,4,5].

Considering bone graft materials that are currently available, autogenic bone grafting provides the best results in terms of bone regeneration. Autogenic bone grafts (autograft) are commonly obtained from a patient’s ilium, ribs, tibia, and similar bones [6,7,8,9]. Autogenic bone grafting is undoubtedly an effective method; however, it could cause surgical damage to other parts of the body [10,11,12]. Moreover, since allogenic bone grafts (allograft) are obtained from cadavers, there is a risk of developing infectious diseases and immunological rejection as well as shortage of graft material supply and high costs. Therefore, heterogeneous bone grafts (xenografts such as bovine bone) and synthetic bone graft materials are widely used. However, in the case of xenografts, variations have been observed in the regeneration ability owing to differences in constituent components. For synthetic bone grafts, hydroxyapatite (HA, Ca_5_(PO_4_)_3_OH) and β-tricalcium phosphate (β-TCP, Ca_3_OPO_4_)_2_) are primarily used owing to their biocompatibility and bioactivity. In the human body, the bone tissue contains hydroxyapatites that act as a reservoir of minerals and contain 99% Ca and 85% P [13,14,15,16]. Therefore, HA and β-TCP are mainly used as synthetic bone graft materials because of the similarity of these components to the bioavailable substances.

Furthermore, the ability of soft tissues to withstand physiological stress is important to rule out distortion of the regenerated bone, and bone block grafts are essential because certain bone grafts cannot resist the stress induced by the nearby soft tissues. In the case of block-type grafts, it is possible to avoid the distortion of the defect space due to the pressure from adjacent soft tissues [17]. Thus, HA and/or β-TCP particles have been extensively studied for developing synthetic bone graft blocks [18,19,20,21,22,23]. However, the block-type bone grafts composed of HA and/or β-TCP are brittle because of their mechanical characteristics, which could result in a sudden decrease in the mechanical stiffness during decomposition in the body [24,25,26,27]. To overcome the brittleness of these ceramic block-type bone grafts, bone graft scaffolds mixed with synthetic biocompatible/biodegradable polymers and osteoconductive ceramic particles (HA and β-TCP) have been fabricated [28,29,30,31]. Many researchers have developed polymer/ceramic composites and also demonstrated their osteoconductive effects such as osteoblast activity and structural the reinforcement [32,33]. However, polymer/ceramic composites have a drawback that ceramic particles exist as inclusions in the substrate (polymer) and cannot act as osteoconductive materials. The osteoconductive effect of ceramic particles is only activated with the exposure of their surfaces to the healing environment. Moreover, composite bone grafts cannot be controlled in terms of their three-dimensional architectures.

After regenerative alveolar using a bone graft, an implant fixture is inserted for the fixation of an artificial tooth. Commercially, titanium and its alloys are considered as standard materials for dental implant fixtures. However, these materials have disadvantages such as the inducible metal allergy and discoloration of the marginal mucosa [34]. In this respect, functional implant fixtures composed of bioinert and biocompatible materials such as polyetheretherketone (PEEK) and zirconia have been widely studied as a possible alternative to titanium implant fixtures [35]. In addition, functional integral designs such as one-piece zirconia implant have been introduced, which can be mounted directly on the alveolar bone [36]. However, the use of these dental fixtures must be preceded by alveolar bone reconstruction.

Here, we propose and fabricate a novel three-dimensionally (3D) printed hybrid scaffold that can be combined with a metal implant fixture and bone graft particles. The 3D printed hybrid scaffold can be used to reduce surgical operation time and to maintain the osteoconductive abilities of the bone graft particles. The hybrid scaffold could be combined with an implant fixture during the first surgical operation, so that the second operation may be avoided. Further, the 3D printed hybrid scaffold could be customized as a frame and designed to fit well in the defect area. Therefore, when using a customized frame during dental implant surgery, the membrane used to immobilize the inserted bone particle graft is rendered unnecessary. Moreover, in this study, the salt leaching using particles (SLUP) was implemented to fill the implant with osteoconductive ceramic particles [37,38]. The exposed surface areas of the osteoconductive ceramic particles were maximized without scattering (loss) of the ceramic particles. If a dental surgeon uses the hybrid scaffold for edentulous patients with severe alveolar bone defects, the surgical procedures could be simplified as shown in Figure 1e–g. 

We evaluated the feasibility of our proposed hybrid scaffold by morphological analysis, porosity distribution analysis, wettability test, CCK-8 assessment, and alkaline phosphatase (ALP) assessment and compared the results with several control groups. For simplicity, the grid-type scaffold and hybrid scaffold without HA/β-TCP were named as control groups Ⅰ and Ⅱ, respectively.

## 2. Results

### 2.1. Morphology Analysis of the Fabricated Hybrid Scaffold

**Figure 2:**
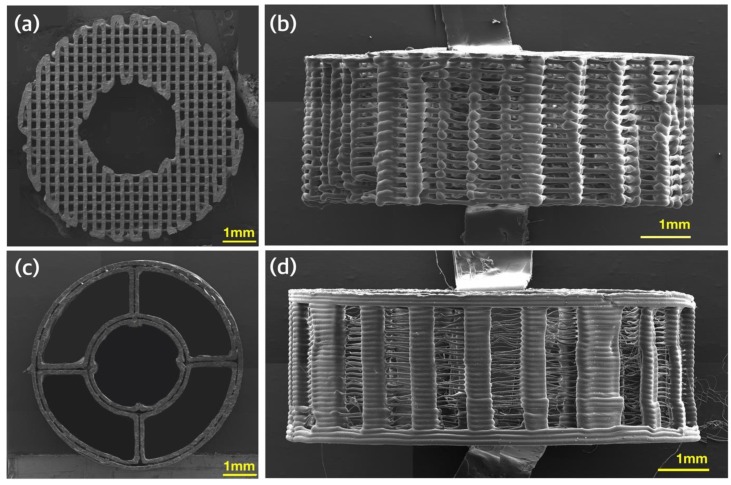
Morphological analysis of three-dimensionally (3D) printed scaffolds using FE-SEM: (**a**) top view of control group Ⅰ; (**b**) side view of control group Ⅰ; (**c**) top view of the polycaprolactone (PCL) frame of the hybrid scaffold; and (**d**) side view of the PCL frame of the hybrid scaffold.

An analysis of the morphology of the fabricated control group Ⅰ using FE-SEM revealed a pore size of approximately 175 to 200 µm and a thickness of approximately 150 µm for the polycaprolactone (PCL) strand, as depicted in Figure 2a. The side view of control group Ⅰ also demonstrated well-defined and interconnected pores, as depicted in Figure 2b. Figure 2c shows the PCL frame of the hybrid scaffold. The vacant internal regions of the hybrid scaffold were designed to be filled with the particle mixture. The thickness of the strand in the PCL frame of the hybrid scaffold was approximately 430 µm. As seen in Figure 2d, the column shape was manufactured well.

**Figure 3:**
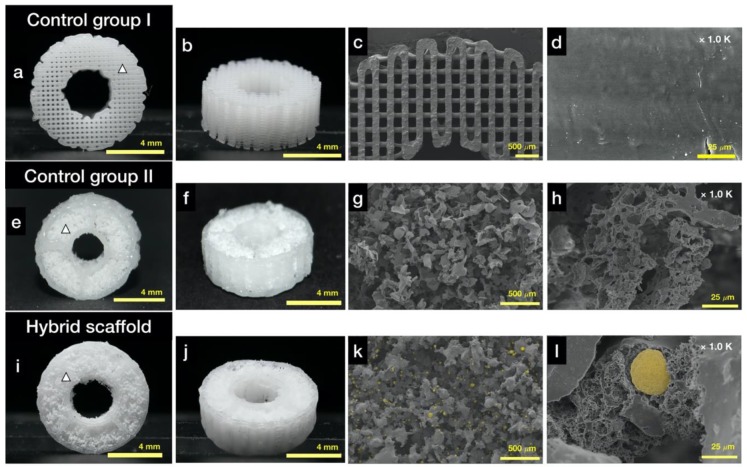
Morphological analysis using optical camera and FE-SEM: (**a**) photograph of the top view of control group Ⅰ (white triangle: position of the Figure 3d); (**b**) photograph of the isometric view of control group Ⅰ; (**c**) FE-SEM image of the top view of control group Ⅰ; (**d**) FE-SEM image of the surface of control group Ⅰ; (**e**) photograph of the top view of control group Ⅱ (hybrid scaffold without HA/β-TCP, white triangle: position of the Figure 3h); (**f**) photograph of the isometric view of control group Ⅱ; (**g**) FE-SEM image of the top view of control group Ⅱ; (**h**) FE-SEM image of the surface of pores in control group Ⅱ; (**i**) photograph of the top view of the hybrid scaffold (white triangle: position of the Figure 3l); (**j**) photograph of the isometric view of the hybrid scaffold; (**k**) FE-SEM image of the top view of the hybrid scaffold (yellow dots: osteoconductive ceramic particles); and (**l**) FE-SEM image of the surface of pores in the hybrid scaffold (yellow dot: osteoconductive ceramic particle).

Figure 3 shows an image of the samples of all experimental groups (grid-type scaffold as control group Ⅰ, hybrid scaffold without HA/β-TCP as control group Ⅱ, and hybrid scaffold with HA/β-TCP). For the analysis of the proposed hybrid scaffold, three experimental groups were formed. Control group Ⅰ was synthesized using the PCL material only in a donut shape with a grid-type inner structure, as depicted in Figure 3a,b. Naturally, the surface of control group Ⅰ was smoother than those of control group Ⅱ and the hybrid scaffold (Figure 3c,d). The scaffold in control group Ⅱ was prepared using a PCL frame and a particle mixture without HA and β-TCP, as depicted in Figure 3e,f. The proposed experimental group, i.e., the hybrid scaffold, was prepared using a PCL frame and a particle mixture following the abovementioned process, as depicted in Figure 3i,j. 

In the case of the hybrid scaffold, the morphological analysis using FE-SEM confirmed the global structure of the pores to be irregular (Figure 3k,l). In addition, HA and β-TCP particles were attached well to the fused PCL particles (yellow areas in Figure 3k,l). Several microsized structures were also observed, as seen in Figure 3l. In the case of control group Ⅱ, which did not contain HA and β-TCP particles, the morphological characteristics were mostly similar to those of the hybrid scaffold, as depicted in Figure 3g,h. In the case of control group Ⅰ, grid-type strands with mostly smooth surfaces were observed.

**Figure 4:**
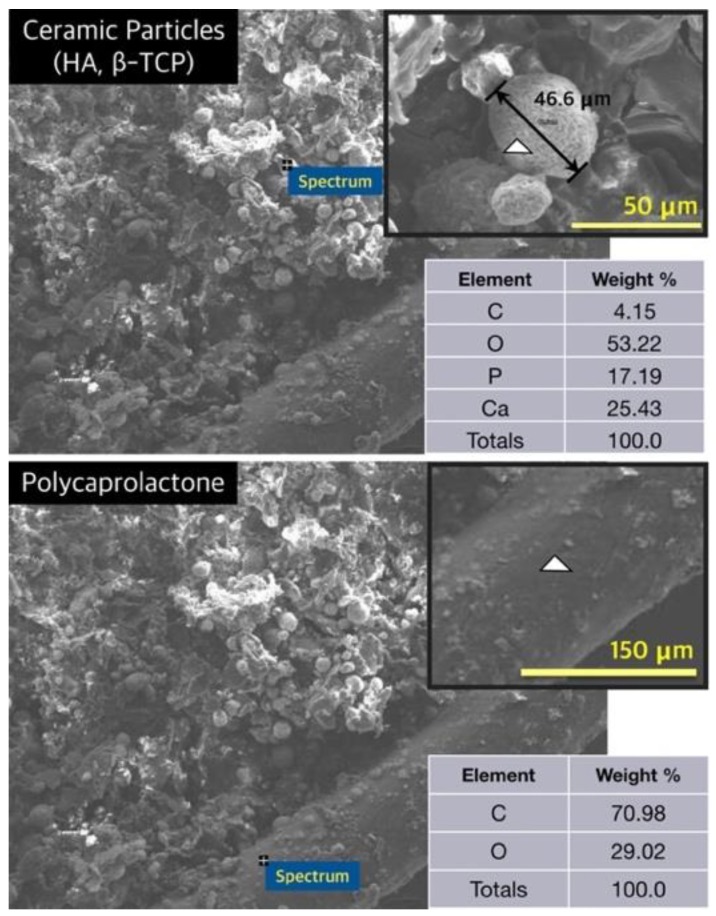
Analysis of energy-dispersive spectroscopy (white triangle: measured point).

The energy-dispersive spectroscopy (EDS) analysis confirmed the exposure of HA and β-TCP particles in the hybrid scaffold, as depicted in Figure 4. The P and Ca components were not detected in the PCL frame; on the contrary, the P and Ca components were detected in particles assumed to be HA and β-TCP (Figure 4).

### 2.2. Characteristics of the Fabricated Hybrid Scaffold

**Figure 5:**
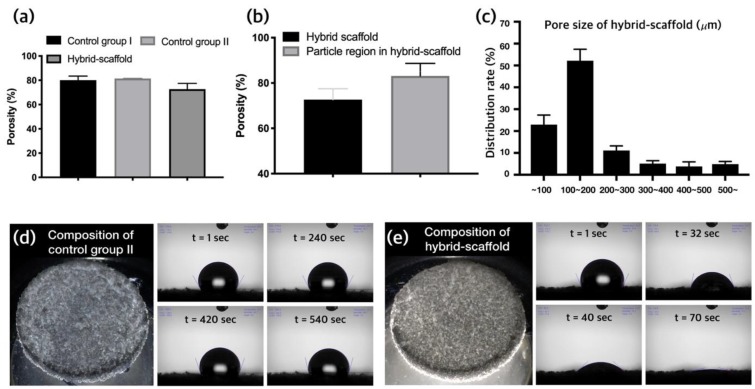
(**a**) Comparison of porosity, (**b**) compared porosity between the hybrid scaffold and the particle region in the hybrid scaffold, (**c**) pore size distribution rate in the hybrid scaffold, (**d**) wettability test of control group Ⅱ, and (**e**) wettability test of hybrid scaffold specimens combined with ceramic materials (HA and β-TCP).

As depicted in Figure 5a, the calculated porosity results of the three groups (control group Ⅰ, control group Ⅱ, and hybrid scaffold) were 80.02% ± 3.32%, 81.42% ± 0.15%, and 72.68% ± 4.79%, respectively. The porosity was calculated using 10 specimens for each group and measurements of the volume percentages. Moreover, the porosity of the particle region, except for the PCL frame in the hybrid scaffold, was 83.14% ± 5.5% (Figure 5b).

The dimensions of the hybrid scaffold was measured as following: inner diameter (D_in_) = 2.67 ± 0.11, outer diameter (D_out_) = 7.47 ± 0.08, and height (h) = 2.99 ± 0.06 mm. The measurements of scaffold dimensions were averaged over five specimens. As mentioned above, the design values of D_in_, D_out_, and h were 3, 8, and 3 mm, respectively.

An analysis of the pore size distribution using the mercury penetration method revealed that the percentage of pores having sizes of 100 to 200 μm was 52% ± 5.2%. The percentage of pores having sizes less than 100 μm was 22% ± 4.4%, whereas the percentage of pores having sizes more than 200 μm was 26% ± 4.4% (Figure 5c).

### 2.3. Assessment of Water Absorption in the Hybrid Scaffold

To estimate the enhancement of cell adhesion ability by the ceramic particles (HA and β-TCP), wettability tests were conducted for control group Ⅱ and hybrid scaffold specimens. It was observed that the water droplet on the specimen made only with the control group Ⅱ material was hardly absorbed. The initial contact angle was 118°, and the contact angle was 116.9° after 540 s (Figure 5d). On the contrary, the initial contact angle for the hybrid scaffold specimen (containing hydrophilic materials HA and β-TCP) was 112.7°, and the drop of water was completely absorbed after 70 s (Figure 5e).

### 2.4. Mechanical Property Analysis

**Figure 6:**
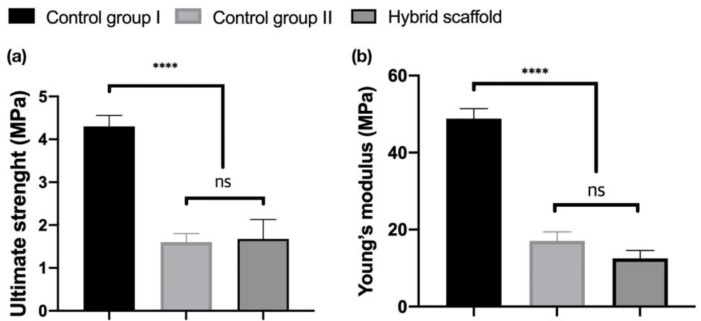
Analysis of the compressive test of each specimen using the ultimate strength (**a**) and Young’s modulus (**b**) (ns: not significant, **** *p* < 0.0001).

Based on the stress–strain curves, the ultimate strengths of all specimens (control Ⅰ, control Ⅱ, and hybrid scaffold) were measured as 4.3 ± 0.3, 1.6 ± 0.2, and 1.675 ± 0.457 MPa, respectively (Figure 6a). The Young’s moduli of all groups were measured as 48.81 ± 2.59, 17.05 ± 2.4, and 12.52 ± 2.04 MPa, respectively (Figure 6b).

### 2.5. In Vitro Cell Viability and Proliferation

#### 2.5.1. Live and Dead Assays

**Figure 7:**
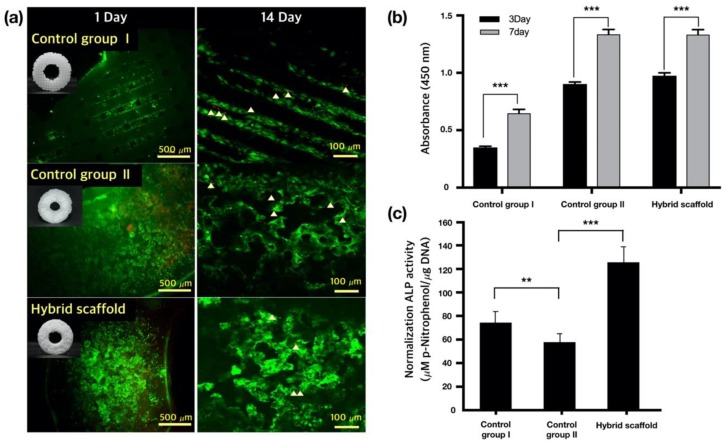
(**a**) Live/dead assays of three groups 1 day (magnification: ×100) or 14 days (magnification: ×200) after cell seeding (white triangle: dead cells), (**b**) CCK-8 assays of the three groups (*** *p* < 0.0005), (**c**) alkaline phosphatase (ALP) activities of Saos-2 cells seeded on different scaffolds 14 days after culture (** *p* < 0.005, *** *p* < 0.0005).

After cell seeding, the cell viability was determined using the live/dead cell staining, as depicted in Figure 7a. The cytoplasms of live cells were stained green, whereas those of the dead cells were stained red. On day one after cell seeding, cell death was observed in control group Ⅱ and the hybrid scaffold, as compared with in control group Ⅰ; however, cell viabilities in control group Ⅱ and the hybrid scaffold were not significantly different from that in control group Ⅰ 14 days after cell seeding.

#### 2.5.2. Cell Proliferation

Cell proliferation in the scaffolds was quantified using the CCK-8 assay, as depicted in Figure 7b. Three days after cell seeding, Saos-2 cells in control group Ⅱ and the hybrid scaffold demonstrated increased proliferations, which was three times that observed in control group Ⅰ. After seven days of culture following cell seeding, no statistically significant difference in cell proliferation was observed between control group Ⅱ and the hybrid scaffold. However, the cell growth rates (for control group Ⅱ and the hybrid scaffold) were twice that observed for control group Ⅰ. Thus, it was confirmed that cells were growing stably in control group Ⅱ and the hybrid scaffold.

#### 2.5.3. ALP Activity of Saos-2 Cells

The ALP activities were analyzed 14 days after seeding of Saos-2 cells on the osteogenic scaffolds. As shown in Figure 7c, the ALP activity of the hybrid scaffold was superior to those of control groups Ⅰ and Ⅱ. These results could be attributed to HA and TCP particles, which are useful for bone formation.

## 3. Discussion

In the present study, we proposed and fabricated a hybrid scaffold for patients requiring alveolar bone regeneration for dental implant surgery. The hybrid scaffold was designed to affix an implant onto a customized 3D printed PCL frame. Moreover, the inside region of the hybrid scaffold could be filled with osteoconductive ceramic particles (HA and β-TCP). These particles were immobilized using fused PCL particles. Furthermore, the SLUP scaffold fabrication process [18] was implemented to form well-interconnected pores using sodium chloride (NaCl) and polyethylene oxide (PEO) particles. As depicted in Figure 1e–g, the use of the proposed hybrid scaffold for dental implant could significantly reduce surgery time, with a concomitant decrease in surgical cost and patient discomfort. In addition, since the frame of the hybrid scaffold is fabricated using 3D printing technology, a customized implantable scaffold having the same size and shape as the defect bone area of the patient can be produced. Further, since the PCL frame protects the loss of bone graft particles, the membrane used to cover the graft site is rendered unnecessary. 

The morphological analysis of the fabricated hybrid scaffold revealed shrinking compared to the designed dimensions of the STL file. It was observed that the shrinkages of D_in_, D_out_, and h from the designed values were 11%, 7%, and 0.5%, respectively. To fuse PCL particles with HA and β-TCP particles for immobilization, an increase in the temperature above the melting point of the PCL material was required. However, since the frame of the hybrid scaffold was also fabricated using the PCL material, increasing the temperature above the melting point would also melt the hybrid scaffold. The standalone PCL frame thus shrank because of excess melting temperature, drastically shrinking the radial dimensions, i.e., D_in_ and D_out_. However, the height dimension shrank negligibly, because solid columns were observed in the height direction. If we use other biocompatible materials with higher melting temperatures (such as PLA) than that of PCL, this shrinkage phenomenon can be prevented. 

As depicted in Figure 3k,l, HA and β-TCP particles were clearly exposed in the hybrid scaffold. These ceramic particles have been employed in bone tissue engineering owing to their chemical structures, which are similar to those of the mineralized constituents of bone [31]. HA and β-TCP particles have excellent physicochemical properties, such as resorbability and bioactivity, as well as an osteoconductive effect from the release of Ca and P ions into surrounding tissues [39,40]. In particular, β-TCP has relatively excellent biodegradable properties than the HA particles. Thus, recently, the use of HA/TCP composites has been widely studied in bone tissue engineering. These HA/TCP composite materials can provide long-term osteoconductive effects as well as initial bioactivity owing to different biodegradable rates [41]. The ALP results verified this ability, as depicted in Figure 7c. The SLUP scaffold fabrication process induced the exposure of HA and β-TCP particles. 

According to the results of the previous study on the SLUP scaffold [18], well-interconnected pores were observed compared with the conventional salt leaching method. In this study, this phenomenon was observed in the SEM images shown in Figure 3g and Figure 4h,k,l. The SEM images demonstrated well-interconnected, random-sized pores that were quantitatively analyzed by porosimetry (Figure 5c). The weight ratio of the particle mixture (PCL:PEO:NaCl:HA:β-TCP) was 1:1:5:0.5:0.5, and these values could be converted to the volume ratio of 19.9:20.1:52.7:3.6:3.7. PEO and NaCl were used as porogens, as their particle forms could be dissolved in water. Therefore, after leaching out with deionized (DI) water, PEO and NaCl were removed, and associated pores were created. According to the volume ratio, the PEO and NaCl volume was 72.8%; however, the porosity of the particle region was calculated to be 83.14% ± 5.5%. This difference could be attributed to intrinsic pores existing between particles. Moreover, we can say that these intrinsic pores contributed to the well-interconnected pores after leaching out PEO and NaCl particles. This structural characteristic is important for the exchange of nutrition and waste in the 3D scaffold. 

According to the water absorption test depicted in Figure 5d,e, we can easily verify that the hydrophilic materials, HA and β-TCP, were exposed in the hybrid scaffold. This enhanced the osteoconductive ability and bone formation. The improved ALP results of the hybrid scaffold are shown in Figure 7c and are one of the advantages of the hybrid scaffold.

In the result of the mechanical analysis using the compressive test, the ultimate strengths and the Young’s moduli were not much difference between control group Ⅱ and hybrid scaffolds (Figure 6a,b). Therefore, ceramic particles with a volume ratio (PCL:Pore:HA:β-TCP= 19.9:72.8:3.6:3.7) of in this study did not contribute significantly to the improvement of mechanical stiffness. However, the comparison of the current results and the previous results for compressive stiffness of the SLUP scaffold (2.80 ± 0.90 MPa) [42] revealed that the existence of the PCL framework considerably improved the mechanical property (12.52 ± 2.04 MPa) of the entire hybrid scaffold. 

## 4. Materials and Methods

### 4.1. Materials

Polycaprolactone (Mn = 45,000, Sigma-Aldrich, St. Louis, MO, USA) was prepared to fabricate a biocompatible frame for a hybrid scaffold. For osteoconductive ceramic materials, HA (particles, CAS no. 12167-74-7, Sigma-Aldrich, St. Louis, MO, USA) and β-TCP (particles, CAS no. 7758-874, Sigma-Aldrich, St. Louis, MO, USA) were prepared. NaCl (CAS no. 7647-14-5, Sigma-Aldrich, St. Louis, MO, USA) and PEO (average Mv: ~300,000, CAS no. 25322-68-3, Sigma-Aldrich, St. Louis, MO, USA) were also prepared. NaCl and PEO were prepared as particles having sizes of 100 to 180 μm with the aid of relevant sieves. Moreover, PCL particles were prepared using a cryogenic grinder (Freezer/mill 6770, SPEX, Metuchen, NJ, USA). The PCL particles having a size range of 63 to 100 μm were also sieved using appropriate-sized sieves. The particle sizes were decided based on the results of our previous study on the SLUP scaffold [42].

The model of the three-dimensional frame of the scaffold was designed using a commercial software (CATIA V5). The weights of the scaffolds were measured using a digital scale (HS204; Hansung, Seoul, Korea) with a 0.1 mg resolution. To study the overall morphology, optical photographs were obtained using an optical microscope (Mi-9100 ZOOM(S), Magic i, Seoul, Korea). Saos-2 cells were obtained from the Korean Cell Line Bank.

### 4.2. Design of the Hybrid Scaffold

**Figure 8:**
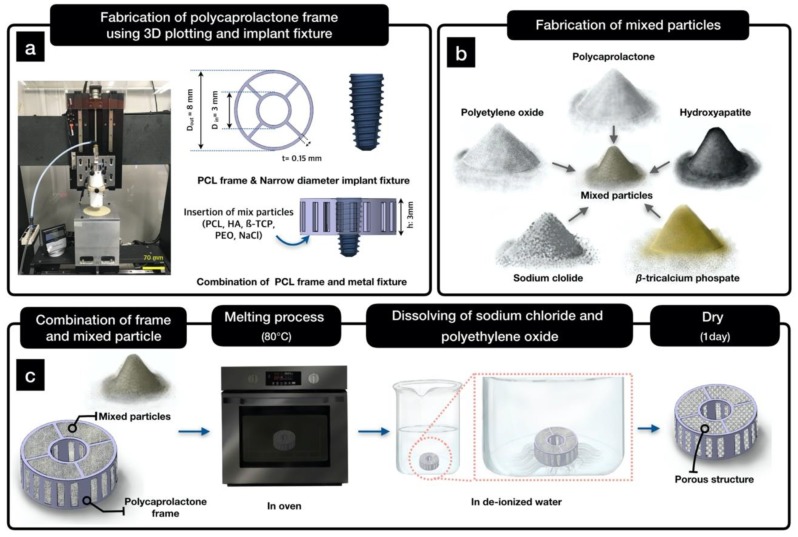
Schematic of the fabrication of a hybrid scaffold: (**a**) design of the hybrid scaffold for narrow-diameter implant fixtures; (**b**) preparation of mixed particles; and (**c**) process of the fabrication of the hybrid scaffold.

The frame of the hybrid scaffold was designed to allow for the insertion of narrow-diameter implant fixtures. The implant fixture had two screw parts, as depicted in Figure 8a. We used the CATIA program to design the three-dimensional frame of the hybrid scaffold. The frame was designed to have an inner diameter (D_in_) of 3 mm, an outer diameter (D_out_) of 8 mm, and a height (h) of 3 mm, as depicted in Figure 8a. Moreover, it was designed to have a donut shape, so that osteoconductive particles could be filled into the frame.

### 4.3. Fabrication of a PCL Frame Using 3D Printing

Open source Slic3r (Version 1.2.9) was used to generate a tool path from the STL file obtained from CATIA. The frame was then fabricated using a laboratory-prepared 3D plotting device, as depicted in Figure 8a. The frame was fabricated using PCL (Mn = 45,000, Sigma-Aldrich) as follows. The PCL pellets were melted in a stainless-steel barrel at 80 °C. A pneumatic pressure of 600 ± 10 kPa and a feed rate of 1 mm/s were used for fabrication.

### 4.4. Fabrication of a Hybrid Scaffold Using Mixed Particles

The prepared PCL, PEO, NaCl, HA, and β-TCP particles were mixed using a stirrer (HS-50 A; Daihan Scientific, Gangwon-do, Korea) for 24 h with a weight ratio of 1:1:5:0.5:0.5, as depicted in Figure 7b. Subsequently, the PCL frame was laid onto a Teflon block, and the particle mixture was poured inside of the PCL frame. Afterward, the Teflon block (with the PCL frame filled with mixed particles) was put into an oven that was operated at 80 °C for 10 min, as depicted in Figure 7c. PCL and PEO particles were slightly melted to prevent scattering of the remaining particle mixture (NaCl, HA, and β-TCP). Moreover, the structure of the PCL frame was maintained under this condition. Subsequently, the fabricated specimen was soaked in DI water to leach out NaCl and PEO, as depicted in Figure 8c. 

For the wettability test, two types of simple cylindrical plate specimens were fabricated to represent the wettability characteristics of control group Ⅱ (hybrid scaffold without HA/β-TCP particles) and the hybrid scaffold. The control group Ⅱ of cylindrical plates having a diameter of 10 mm and a height of 3 mm was fabricated following the abovementioned process for mixing the particles, melting, and leaching out without HA/β-TCP particles. The hybrid scaffold specimens of cylindrical plates with the same dimensions were fabricated as well.

### 4.5. Characterization of the Fabricated Hybrid Scaffold

To observe the surface and internal pores and osteoconductive ceramic of the fabricated hybrid scaffold, FE-SEM (S-4800; Hitachi, Tokyo, Japan) was used. Fabricated hybrid scaffolds were coated with platinum for 60 s before the SEM analysis. The SEM images were captured using a voltage of 5.0 kV. In addition, we used EDS (S-4800; Hitachi, Japan) to confirm the existence of ceramics (HA and β-TCP) inside the fabricated hybrid scaffold. The porosity of the hybrid scaffold was defined using Equations (1)–(3):

(1) $$Porosity\left(\%\right)=\frac{V_{0}-\left(V_{F}+V_{P}\right)}{V_{0}}\times 100,$$

(2) $$V_{F}=\frac{m_{frame}}{\rho_{PCL}},$$

(3) $$V_{P}=\frac{m_{PCL}}{\rho_{PCL}}+\frac{m_{HA}}{\rho_{HA}}+\frac{m_{TCP}}{\rho_{TCP}},$$

where *V*_0_ refers to the apparent volume of the fabricated hybrid scaffold, *V_F_* is the volume of the PCL frame, and VP is the volume of the filled particle in the hybrid scaffold after leaching out, respectively; and $\rho_{PCL}$, $\rho_{HA}$, and $\rho_{TCP}$ are the densities of PCL, HA, and β-TCP, respectively; The masses of the PCL particle, HA particle, and β-TCP particle, which were filled into the hybrid scaffold, are denoted by mPCL, mHA, and mTCP, respectively. The porosity was calculated by averaging the porosity values of five specimens.

The pore distribution in the hybrid scaffold was measured by porosimetry (MicroActive AutoPore V 9600; Micromeritics, Norcross, GA, USA). The mercury penetration method was used, and the average value of five specimens was calculated.

For the wettability experiment, a water contact angle meter (SmartDrop; Femtofab, Gyeonggi-do, Korea) was used. Five milliliters of water were dropped on control group Ⅱ and hybrid scaffold specimens, and contact angles were recorded at the initial stage and after 540 s.

### 4.6. Compressive Test

Compressive tests using a Universal Test Machine (UTM; Model E42, MTS, Berlin, Germany) were performed to analyze mechanical properties such as the ultimate strength and the Young’s modulus of all the fabricated specimens. Five specimens were prepared in each group. The compressive test condition used was as following: capacity of 5.0 kN load cell and test speed of 1 mm/min. 

### 4.7. In Vitro Cell Characterisrics

#### 4.7.1. Cell Culture

Saos-2 cells (human osteogenic sarcoma cells; Korea Cell line Bank, Seoul, Korea) were cultured in Dulbecco’s Modified Eagle Medium (DMEM, Gibco, New York, NY, USA) supplemented with 10% fetal bovine serum (FBS, Gibco), 2 mM L-glutamine, 100 U/mL penicillin, and 100 µg/mL streptomycin (Gibco) and maintained in a humidified incubator at 37 °C with 5% CO_2_. The medium was changed every other day, and proliferated cells were split using 0.05% trypsin with 0.02% EDTA (Sigma-Aldrich), when the assay was performed. To measure the cell characteristics of Saos-2 cells using scaffolds, cells in suspension were seeded into each scaffold at a density of 1 × 10^5^ cells/20 μL media and incubated overnight. The cell-seeded scaffolds were maintained in an incubator for up to 14 days to evaluate the ALP activity.

#### 4.7.2. Live and Dead Assays

To evaluate the cell viability in the scaffold, cells were stained using the live/dead assay kit (Calcein-AM, dead: ethidium homodimer-1, Ethd-1, Thermo Fisher Scientific, Waltharm, MA, USA). The cells in the scaffolds were washed twice with phosphate-buffered saline (PBS, Gibco). Subsequently, the cells were exchanged with the live/dead assay solution and incubated for 30 min at room temperature. The stained cells were then photographed using a confocal microscope (FV1200, Olympus, Tokyo, Japan).

#### 4.7.3. Cell Proliferation Assay

Cell proliferation was determined using a cell-counting kit (CCK-8, Invitrogen, Carlsbad, CA, USA). Briefly, cells were seeded into each scaffold at a density of 1 × 10^5^ cells/scaffold and cultured for 7 days. Cells in the media at 3 days or 7 days were then treated with the CCK-8 solution and incubated for 4 h at 37 °C, following which the absorbance was measured at 450 nm using a microplate reader.

#### 4.7.4. Alkaline Phosphatase Assay

The cells were seeded into the scaffolds, and at 14 days, the ALP activity was measured using the TRACP & ALP assay kit (TaKaRa-Bio Inc., Kusatsu, Japan). The samples were washed with PBS and incubated with an extraction buffer. Fifty microliters of lysate were mixed with 100 µL of the ALP substrate solution containing p-nitrophenyl phosphate (pNPP) with 0.2 M Tris-HCl (pH 9.5) and 1 mM MgCl_2_ at 37 °C for 20 min. The reaction was stopped by 50 µL NaOH, and the ALP activity was determined spectrophotometrically at 405 nm using a microplate reader. The total DNA concentration was measured using a DNA assay kit. The ALP activity was expressed as µmol of p-nitrophenol formed per milligram of the total DNA.

### 4.8. Statistical Analysis

The data are expressed as the mean ± standard deviation. All statistical analyses were conducted using GraphPad Prism (GraphPad software; La Jolla, CA, USA). All experiments were performed in triplicate. Statistical significance was determined using two-tailed t-tests. Statistical significances were set as following: ** *p* < 0.005, *** *p* < 0.0005, and **** *p* < 0.0001.

## 5. Conclusions

In the present study, a hybrid scaffold was designed and fabricated. It combined both bone grafts (HA and β-TCP) and implant fixtures to simplify the alveolar bone regeneration and dental implant processes. The fabricated hybrid scaffold had excellent wettability, which can be interpreted as initial cell adhesion ability, owing to the exposure of HA and β-TCP particles. Moreover, the in vitro cell culture assays using Soas-2 cells demonstrated excellent cell culture characteristics. The ALP results verified the bone regeneration ability with and without osteoconductive ceramic particles.

As the future work, we plan to analyze vertical bone formation and histological analysis by animal experiments. In addition, the coating of bone morphogenetic protein 2 is planned along with the use of ceramic particles to determine the possibility of maximizing bone formation. We believe that the use of hybrid scaffolds in clinical practice could significantly overcome the drawbacks of block-type ceramic bone grafts. In addition, as implant fixtures and bone grafts could be inserted into one dental structure, it could be considered a versatile method to simplify the process of the existing implant surgery.

## Abbreviations

PCL	polycaprolactone
PEO	polyethylene oxide
HA	hydroxyapatite
β-TCP	β-tricalcium phosphate
NaCl	sodium chloride
SLUP	salt leaching using particles
ALP	alkaline phosphatase assay
PBS	phosphate-buffered saline
